# Microwave Modification of an Epoxy Basalt-Filled Oligomer to Improve the Functional Properties of a Composite Based on It

**DOI:** 10.3390/polym15092024

**Published:** 2023-04-24

**Authors:** Amirbek Bekeshev, Ekaterina Vasinkina, Svetlana Kalganova, Yulia Kadykova, Anton Mostovoy, Andrey Shcherbakov, Marina Lopukhova, Zukhra Aimaganbetova

**Affiliations:** 1 Laboratory of Polymer Composites, K. Zhubanov Aktobe Regional State University, Aliya Moldagulova Avenue 34, Aktobe 030000, Kazakhstan; amirbek2401@gmail.com; 2 Department of Electric Power and Electrical Engineering, Yuri Gagarin State Technical University of Saratov, Polytechnichskaya St., 77, 410054 Saratov, Russia; vasinkina1987@mail.ru (E.V.); s.kalganowa2016@yandex.ru (S.K.); kadykova06@yandex.ru (Y.K.); 3 Laboratory of Modern Methods of Research of Functional Materials and Systems, Yuri Gagarin State Technical University of Saratov, Polytechnichskaya St., 77, 410054 Saratov, Russia; 4 Laboratory of Support and Maintenance of the Educational Process, Yuri Gagarin State Technical University of Saratov, Polytechnichskaya St., 77, 410054 Saratov, Russia; 5 Department of Economics and Humanitarian Sciences, Yuri Gagarin State Technical University of Saratov, Polytechnichskaya St., 77, 410054 Saratov, Russia; 6 Department “Physics”, K. Zhubanov Aktobe Regional State University, Aliya Moldagulova Avenue 34, Aktobe 030000, Kazakhstan

**Keywords:** epoxy basalt-filled oligomer, modification, microwave electromagnetic field, epoxy basalt-filled polymer composite material, physicochemical and mechanical properties

## Abstract

The purpose of this work is to study the influence of the electric field strength of an electromagnetic wave with the maximum modifying effect on an epoxy basalt-filled oligomer, which is of great scientific and practical importance for the development of microwave oligomer modification technology. The optimal modes of microwave modification, under which the highest values of the mechanical properties of an epoxy basalt-filled polymer composite material are obtained, are identified: power of 400 W and an exposure time of 24 s. At the same time, the breaking stress in bending increases by 20%, the impact strength increases by 2 times, and hardness increases by 31%. A slight increase of 4.5% in heat resistance is noted compared to the composite obtained on the basis of an oligomer unmodified in the microwave electromagnetic field. The results of resistance to various aggressive environments are obtained, which show that the studied physical and mechanical characteristics of the epoxy basalt-filled material after exposure to an aggressive environment decrease by less than 14%, which corresponds to their good resistance to an aggressive environment. It is established that the effect of the microwave electromagnetic field on an epoxy basalt-filled oligomer is an effective modification method that improves physical and mechanical characteristics with a high level of temporal stability to climatic influences, with a coefficient of property retention of more than 90%.

## 1. Introduction

Composite materials that are affected by high strength and vibration loads during operation due to climatic factors and elevated temperatures are widely used in products and structures in aviation and rocket and space technology, in shipbuilding, rail and road transport, construction and other industries. At the same time, the rapid development of military and space-rocket equipment and technologies requires the creation of more durable, heat- and chemical-resistant materials with complex improved functional properties [1,2,3].

In order to meet the needs of industry and expand the areas of application of polymeric materials, it is advisable to modify already known polymers, imparting various functional properties for directly changing and controlling their structure and their physicochemical and mechanical characteristics. Thus, to impart elastic properties to polymeric materials, they are modified by adding plasticizers [4,5,6]. The addition of fillers makes it possible to increase the strength of epoxy composites, giving them specific physicochemical properties [7,8,9,10,11]. Epoxy polymer composites filled with heat-conducting nanofillers can provide thermal conductivity properties with electrical insulating properties [12]. The addition of 50% by weight carbide fillers significantly increases strength and modulus of elasticity during compression of epoxy composites with a decrease in shrinkage and an increase in adhesion to steel (at normal fracture) [13]. The addition of flame retardants makes it possible to create durable fire-resistant and strong epoxy resins with well-preserved thermal and optical properties [14,15,16].

**Figure 1:**
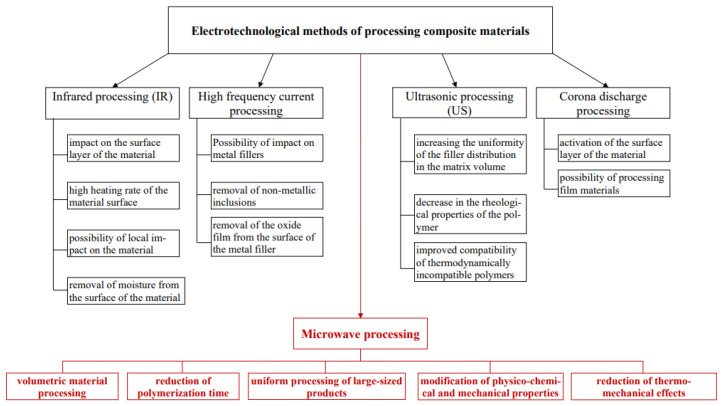
Electrophysical methods for processing composite materials based on epoxy resins.

In order to impart new functional properties to the material, various electrophysical processing methods, such as elastic vibrations of the ultrasonic frequency range, high and ultrahigh frequency currents, corona electric arc, infrared processing and others, are widely used (Figure 1) [17,18,19,20].

One of the methods of electrophysical modification is treatment with high frequency currents. Metal powders are widely used in polymeric materials; high-frequency currents are used to anneal the filler. Annealing of the metal filler is carried out to remove non-metallic inclusions as well as the oxide film formed on the surface of metals. The treated filler readily enters into a chemical reaction with a polymer binder forming chemical bonds, which contributes to an increase in the entire complex of properties of the filled polymer [21].

Ultrasonic treatment makes it possible to evenly distribute the filler, including nanosized particles, in the volume of the matrix to reduce rheological properties of the binder and to improve compatibility of thermodynamically incompatible polymers. This method of treatment also contributes to the collapse of air bubbles formed during the mixing of the filler with the matrix. During the treatment, the energy concentration of ultrasonic vibrations in very small volumes can cause such phenomena as the breakage of the chemical bonds of macromolecules, the initiation of chemical reactions, the erosion of surfaces of solids, etc. [22,23].

In order to increase the reactivity of the polymer material, in many cases a corona discharge is used for thermoplastic polymers [24]. In the corona discharge zone, molecular bonds on the surface of the polymer are broken, and various reactive intermediate oxygen-containing functional groups are formed. It is these groups that effectively increase the chemical interaction of the polymer with the filler. During corona treatment, a corona is formed with the release of ozone (O_3_), which makes it possible to slightly destroy the surface layer of amorphous film polymers in order to form free atoms ready to enter into a chemical reaction, thus increasing the adhesive ability of the material.

The use of infrared processing is advisable for fibrous fillers in order to remove lubricants from their surface. Thus, the lubricant is removed from the surface of reinforcing fillers by annealing at temperatures up to 200 °C. In addition, the use of infrared processing of polymer coatings makes it possible to reduce porosity while improving the quality of coatings [25].

In recent years, technologies related to the processing of polymeric materials in the microwave electromagnetic field (EMF) have been developed [26,27,28]. Studies of electrophysical methods of processing polymers and products have shown the efficiency of using the energy of a microwave electromagnetic field for the modification of polymer composite materials (PCM) based on thermosetting binders. The main advantage of microwave modification is a uniform, volumetric processing of PCM of various shapes and dimensions, which allows significantly speeding up the modification process compared to other processing methods and at the same time results in increasing the quality of finished products and reducing thermo-mechanical effects [29,30,31,32,33].

Processing in a microwave EMF makes it possible to achieve a uniform modification of the polymer without local overheating, which avoids the destruction of the material. The high productivity of the microwave modification of polymers is explained by the fact that non-thermal modification of an object in a microwave EMF is achieved in a much shorter time than under other electrophysical effects [29,30]. 

Works on the processing of dielectrics by the energy of a microwave EMF have been carried out since the middle of the last century. The scientific works of a number of scientists were focused on the thermal effect of a microwave EMF on dielectrics [30,31,32], as well as on the modifying “non-thermal” and “combined” effects of a microwave EMF on polymer dielectrics [31]. In recent years, positive results have been obtained, proving the effectiveness of using microwave EMF for modifying cured epoxy composite polymers in order to reduce internal stresses in a polymer composite material (PCM) [32]. Thus, it can be stated that all research in the field of the microwave modification of polymers is carried out in the following areas:

- The modifying effect of microwave EMF affects the “oligomer-hardener” system, i.e., electrophysical impact directly affects the curing process;
- Microwave heat treatment of a cured epoxy composite.

In this regard, a new scientific task is stated in this scientific work: to establish the modifying effect of a microwave EMF on the “oligomer-filler” system in order to improve the functional properties of the polymer composite material based on it. New electrophysical possibilities for obtaining a modified epoxy basalt-filled polymer composite material with a given set of properties characterize the relevance of scientific research and developments in the field of polymer matrix composite technology.

## 2. Materials and Methods

### 2.1. Materials

In this scientific work, the effect of a microwave electromagnetic field on an epoxy basalt-filled oligomer (EBO) and, as a result, on the properties of an epoxy basalt-filled polymer composite material (EB PCM), was studied.

**Figure 2:**
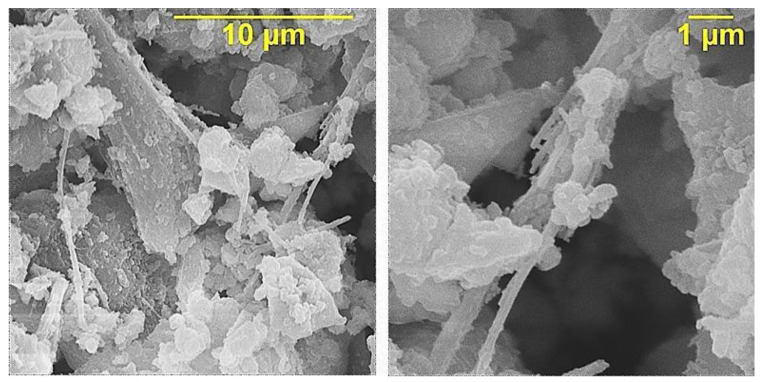
SEM of basalt particles.

Epoxy resin ED-20 manufactured by CHIMEX Limited (St. Petersburg, Russia) was used as a binder for obtaining polymer composite materials. Trichloroethyl phosphate (TCEP) manufactured by Xuancheng City Trooyawn Refined Chemical Industry Co. (Beijing, China) was used as a plasticizer and flame retardant. Trichloroethyl phosphate-tris-(2-monochloroethyl) phosphate (C_6_H_12_Cl_3_O_4_P) is a complete ester of phosphoric acid and ethylene chlorohydrin. It is an effective flame retardant that significantly improves the firefighting properties of materials. Trichloroethyl phosphate forms a homogeneous physical mixture with polymers and does not enter into a chemical reaction with them, which enhances the flame retardant effect. In addition, trichloroethyl phosphate is a good plasticizer. The presence of chlorine atoms in the composition of trichloroethyl phosphate does not reduce its compatibility with polymers. When trichloroethyl phosphate is added to the composition, a self-extinguishing material is obtained, which quickly goes out after the termination of an open flame [33]. Crushed basalt rubble with a particle size of ≤140 µm was used as a filler for epoxy composites. The SEM data of basalt particles are presented in Figure 2. Basalt is an igneous rock solidified in the upper layers of the earth crust. It has high strength and density as well as high chemical properties, fire resistance, strength, durability, sound and heat insulation performance [34].

The choice of basalt as a filler is related not only to its availability, but also to a certain chemical composition: the presence of metal oxides (oxides of iron, calcium, aluminum and titanium) will allow the use of basalt as a fire retardant for epoxy polymers. The chemical composition of basalt was determined on an X-ray analytical microprobe-microscope PAM 30-μ (Scientific instruments, St. Petersburg, Russia) (Table 1).

Taking into account the properties of basalt, it can be assumed that its addition into the polymer composition will provide an increase in physicochemical and mechanical properties of polymer composites. 

The mass content of components in EBO is 100 parts by mass of ED-20 + 40 parts by mass of TCEP + 50 parts by mass of basalt. To obtain an epoxy basalt-filled polymer composite material, polyethylenepolyamine (PEPA) manufactured by CHIMEX Limited (St. Petersburg, Russia) was used as a hardener. Table 2 shows the main properties and characteristics of epoxy resin, TCEP, basalt and a hardener.

### 2.2. Technique for Preparing Samples of Epoxy Basalt-Filled Oligomer for Processing in a Microwave Electromagnetic Field

**Figure 3:**
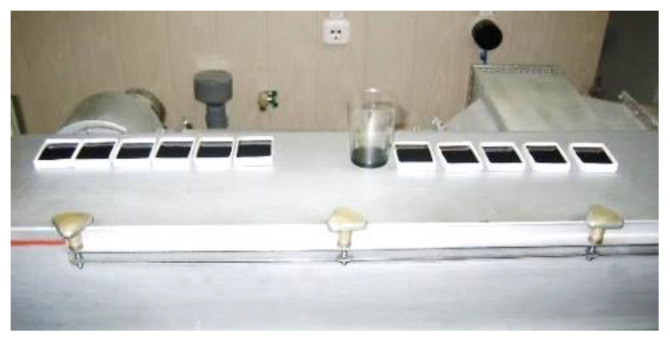
Samples of the epoxy basalt-filled oligomer prepared for processing in a microwave EMF.

To study the effect of microwave EMF modes on EBO, the composition was prepared in a mass ratio: 100 parts by mass of ED-20 + 40 parts by mass of TCEP + 50 parts by mass of basalt. The mixture was thoroughly mixed and poured into cuvettes with the size of 500 × 400 × 100 mm^3^ (Figure 3). The size of the cuvettes was determined by the dimensions of the working microwave chamber and the conditions for the uniform processing of the object. Fluoroplastic which has the properties of a radio-transparent material for a microwave EMF was used as the cuvette material.

**Figure 4:**
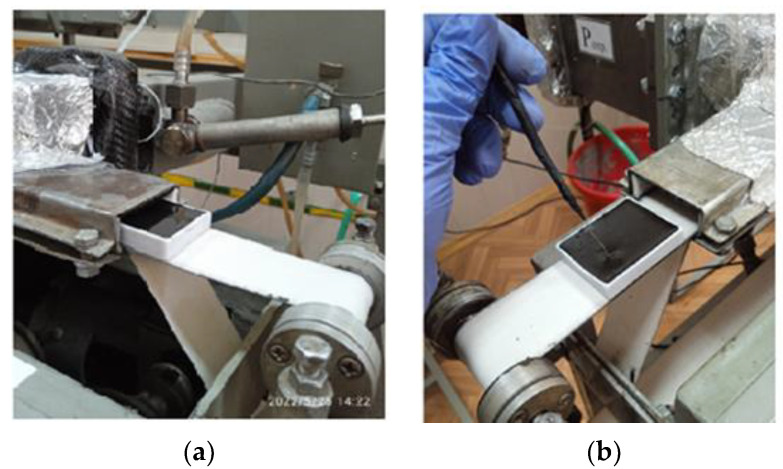
Sections of loading (**a**) and unloading (**b**) the EBO on a conveyor belt through airlock compartments.

The cuvette with the EBO was placed on a conveyor belt in front of the airlock compartment of the microwave installation. After the installation had been switched on, the cuvette was automatically transported to the working chamber, where microwave processing was carried out directly under the specified modes (Figure 4).

### 2.3. Equipment for Experimental Research

**Figure 5:**
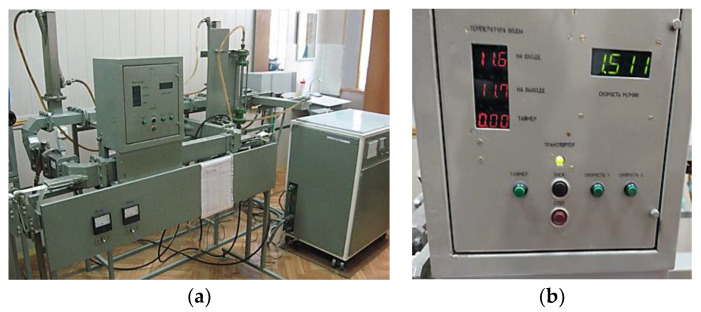
Automated specialized microwave installation for scientific research: (**a**) general view; (**b**) control panel.

The study of the effect of microwave EMF modes on the EBO was carried out using an automated specialized microwave installation for scientific research based on a traveling wave camera (TWC), which allowed controlling the power level and the duration of the object processing (Figure 5).

The installation was designed for microwave processing of liquid, viscous and solid polymeric materials during manual loading and unloading of the object. The working chamber was the main element of the installation since the microwave effect on the processed object takes place in it. A traveling wave chamber (TWV) is a segment of a rectangular waveguide with a 45 × 90 mm^2^ section and waveguide turns at its ends. The TWC operates in a mode similar to that of a traveling wave, which differs favorably from a chamber with a standing wave with the possibility of obtaining better agreement with the microwave generator, i.e., greater efficiency and greater uniformity of processing.

**Figure 6:**
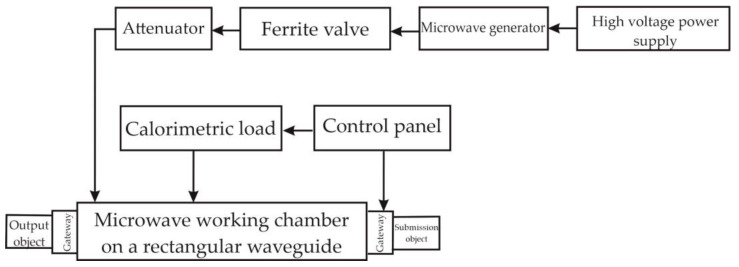
Functional diagram of the automated microwave installation based on a traveling wave camera.

The main elements of the installation were a source of microwave energy, a ferrite valve, an attenuator, a working microwave chamber with a conveyor, a calorimetric load for measuring the transmitted power and a control panel for the conveyor electric drive (Figure 6).

The source of microwave energy was assembled on the M-147 magnetron with a maximum power of 3 kW and a frequency of 2450 MHz. The power supply was equipped with a rectifier and a filter, which made it possible to ensure a stable continuous operation of the magnetron. The selection of the optimal frequency of the microwave exposure to the processed object is of independent scientific interest; however, the frequency of 2450 MHz was chosen for the following reasons:

- It is one of the frequencies allowed by international agreements and the most commonly used in technological installations;
- Application of other frequencies (433 and 915 MHz) would require the use of larger amounts (expenses) of samples at this stage of research.

To ensure reliable operation of the magnetron (stable output power, no frequency pulling), a ferrite valve was used at the magnetron output, which decouples the microwave generator and the microwave module by the reflected wave.

The microwave power regulation in the installation was carried out smoothly using a thyristor converter and was recorded by the value of the anode current. Moreover, the level of the microwave power in the path can be controlled using a variable attenuator assembled on the basis of coupled rectangular waveguides with a 45 × 90 mm^2^ section with 2 ballast calorimetric loads in the side waveguide.

A coaxial calorimetric load was connected to the turn at the output of the working chamber, which made it possible to measure the power transmitted through the working chamber by the calorimetric method:

(1) $$P_{tran}=70G_{\Delta }T$$

where *P_tran_* is the power not absorbed by the object; W, *G* is the water consumption, l/min; and *ΔT* is the temperature difference at the inlet and outlet of the hydraulic system of the calorimetric load. To carry out these measurements, the installation was equipped with a water flow meter, thermocouples that measure the temperature at the inlet and outlet of the ballast calorimetric load with an appropriate indication system in the range from 0 to 100 °C. The entire working chamber was pierced by a transport radio-transparent tape so that the object located on it was in the middle of the wide wall of the waveguide. The conveyor belt was driven by an adjustable electric drive that allowed us to change the speed of the belt. While performing experimental studies, the microwave installation allowed us to establish the value of the generated power and the transmitted that was not absorbed by the object power, as well as to calculate the amount of power absorbed by the object during the microwave processing according to the formula:

*P_abs_* = *P_microwave_* − *P_trans_*. (2)

The time of microwave processing was controlled by the speed of the conveyor belt; the value of the generated power (*P_micr_*) was set according to the magnitude of the anode current of the magnetron and the magnitude of the attenuation of the variable attenuator. The power *P_trans_* that was transmitted through the working chamber and was not absorbed by the EBO was measured by the calorimetric method.

When conducting research using the microwave installation, it is necessary to know the exact values: generated power, transmitted power and reflected power. This is due to the fact that the modifying effect on the EBO is the magnitude of the electric field strength E of the electromagnetic wave, which depends on the absorbed power *P_abs_*. When calculating the absorbed power *P_abs_*, it is necessary to know the exact values of the input and output parameters, the power source for measuring the anode current and the calorimetric load.

### 2.4. Testing of the Composites

Test method for static bending. Tests of samples for resistance to bending loads were carried out using a universal electromechanical testing machine WDW-5E of Time Group Inc. (Beijing, China) at a test speed of 50 mm/min. Bending stress and flexural modulus were determined in accordance with ISO 178:2019. The tests were carried out on samples in the form of blocks with a thickness of 4 mm, a width of 10 mm and a length of the working part of 80 mm.

Brinell hardness test. Brinell hardness was determined in accordance with ISO 2039-1:2001 using an electronic Brinell hardness tester HBE-3000A Beijing United Test Co., Ltd. (Beijing, China).

Method for determining impact strength. Impact strength was determined according to ISO 179-1:2010 using an LCT-50D impact test machine (Beijing United Test Co., Ltd., Beijing, China).

Method for determining water absorption. Water absorption was determined in accordance with ISO 62:2008. The samples were immersed into distilled water at a temperature of (23 ± 2) °C for (24 ± 1) h. The mass of water absorbed by each sample was calculated from the difference between the mass of the sample before and after the test, expressed as a percentage of the initial mass.

Method for determining the oxygen index. The oxygen index of the samples was determined according to ISO 4589-84. The method for determining the oxygen index consists of finding the minimum oxygen concentration in the flow of an oxygen–nitrogen mixture, at which an independent combustion of a vertically located sample ignited from above is observed.

Method for determining the resistance to the action of the chemical environment. The resistance of polymer composite materials to the action of the chemical environment was determined in accordance with ISO 175:2010. The samples were completely immersed into the test liquid for a given time and at a given temperature. The properties of the samples were determined before the immersion and after the removal from the test liquid.

Test methods for aging under the effect of natural and artificial climatic factors. The samples were exposed to natural climatic factors at climatic stations for a given duration of testing. The resistance to the specified effect was determined by changing one or more property indicators (physical–mechanical, electrical, optical, appearance, etc.). The property retention coefficient (*K_t_*) was determined by the formula:

(3) $$K_{t}=\frac{P_{t}}{P_{0}}\cdot 100$$

where *P_t_* is the value of the indicator after testing by the time *t*, and *P*_0_ is the value of the indicator before testing.

Methods for determining density of the displaced volume of liquid. For testing, we used samples with a volume of 1 cm^3^ and a mass of up to 5 g and weighed them in air. After that, the sample was placed into a beaker with distilled water until it was completely immersed without touching the walls and the bottom of the beaker. We made sure that there were no air bubbles on the sample and then weighed it. Density was calculated by the formula:

(4) $$p=p_{W}\cdot \frac{a}{a+w_{1A}-b_{1A}}$$

where $p_{W}$ is the density of distilled water at a temperature of 23.0 °C, equal to 997.5 kg/m^3^; a is the mass of the sample; g is the weight of the wire-suspension (with a load, if it was used) in distilled water; and g is the mass of the sample with the wire-suspension e (and a load, if it was used) in distilled water, g.

Method for determining heat resistance by Vicat. Vicat heat resistance determination was carried out in accordance with the ISO 306:2013 method B50—load 50 N; temperature rise rate was 50 °C/h.

Thermogravimetric analysis of polymers. The thermal stability of the samples was determined by thermogravimetric analysis using a Q-1500D derivatograph (MOM, Budapest, Hungary) of the Paulik–Paulik–Erdey system under the following experimental conditions: a weighed portion was 100 mg, medium, air; heating interval was up to 1000 °C, heating speed was 10 °C/min, and the relative error did not exceed 1%.

Determination of the mass loss of the sample upon ignition in air. To determine the mass loss during ignition in air, samples 35 ± 1 mm wide, 150 ± 3 mm long and 4 ± 1 mm high were made. Preliminarily weighed (with an accuracy of 0.0001 g) samples were suspended vertically in the center of a metal tube so that the end of the sample protruded 5 mm and was 10 mm above the gas burner. A gas burner with a flame height of 40 ± 5 mm was placed under the sample in its center. After 2 min of exposure to the flame, the source of ignition was removed, and the sample continued to burn or smolder on its own. After cooling to room temperature, the sample was weighed (with an accuracy of 0.0001 g), and the mass loss was determined as a percentage of the original sample mass according to the formula:

(5) $$\Delta m=\left(m_{1}-m_{2}\right)\cdot \frac{100}{m_{1}}$$

where *m*_1_ was the mass of the sample before testing, and *m*_2_ was the mass of the sample after testing.

Determination of curing kinetics. When studying the curing kinetics of epoxy compositions, the change in the temperature of the curing process was controlled at a temperature scanning rate of 1 deg·min^−1^. The gelation time corresponding to the time of a sharp rise in the temperature of the curing process and the curing time corresponding to the time of reaching the maximum value of the curing temperature were determined.

The infrared spectroscopy (FTIR) method. IR spectra of the polymer were obtained using a Shimadzu IRTracer-100 (Tokyo, Japan).

Scanning electron microscopy method. The Aspex EXlorer desktop scanning electron microscope is designed to study metal and dielectric samples by detecting backscattered electrons and secondary electrons, as well as characteristic X-ray radiation. During our research, the surface, the chip and the section of samples of epoxy basalt-filled PCM were studied. 

Calculation of the electric field strength of an electromagnetic wave. To calculate the electric field strength of an electromagnetic wave *E*, which depends on the specific absorbed power *P_abs_*, EMF frequency *f*, dielectric permittivity and dielectric loss tangent *tgδ* of PCM, the following relations were used:

(6) $$E=\sqrt{\frac{P_{sp}}{0.278\;\times \;10^{-12}f\varepsilon^{\prime }tg\delta }}$$

where specific power *P_sp_* was determined by the formula:

(7) $$P_{sp}=\frac{P_{abs}}{V}$$

f is the frequency 2450 × 10^6^ Hz.

## 3. Results

### 3.1. Choice of Modes of Microwave Electromagnetic Field Effect on the Basalt-Filled Epoxy Oligomer

**Figure 7:**
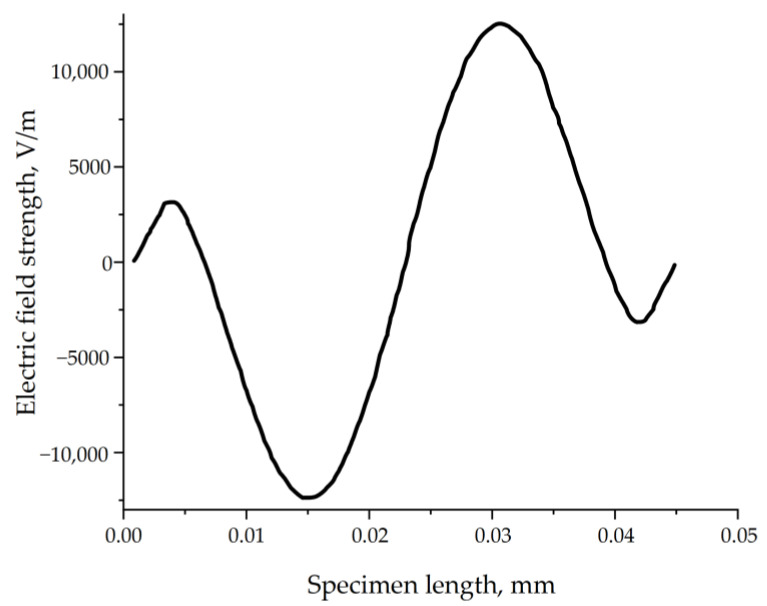
Changing in the electric field strength E of the electromagnetic wave in the EBO sample.

In the COMSOL multiphysics software, the change in the strength E of the electric field of the electromagnetic wave in the EBO sample was obtained, which proves that the processing of the oligomer in the methodical mode in the microwave chamber with a traveling wave makes it possible to achieve its uniform modification (Figure 7).

**Figure 8:**
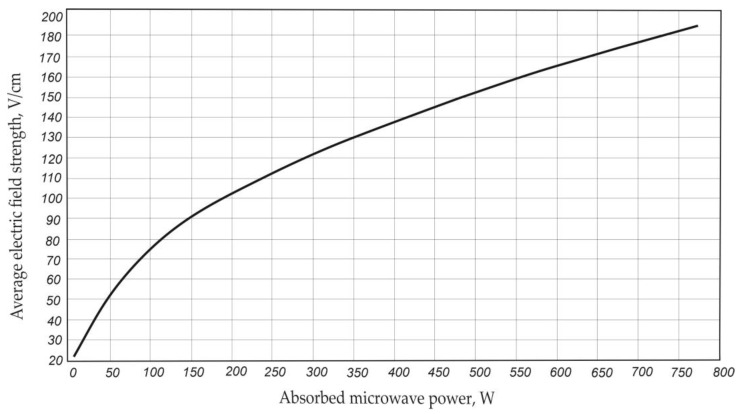
Average values of the electric field strength E of the electromagnetic wave, depending on the microwave power absorbed by the oligomer.

In addition, the average value of strength E of the electric field of the electromagnetic wave (Figure 8) was calculated depending on the microwave power absorbed by the oligomer *R_sp_*.

While conducting our research, we obtained the effect of the generated microwave power *P_micr_* (further referred to as microwave power) on the absorbed microwave power *P_abs_* with the epoxy basalt-filled oligomer on the temperature of the EBO after microwave exposure. The values of the strength of the microwave electromagnetic field during processing of the polymer material were calculated, which have important scientific and practical significance for the development of technology for microwave modification of the oligomer (Table 3).

**Figure 9:**
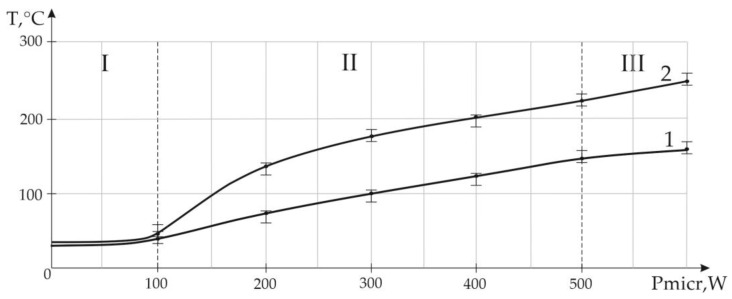
The effect of the generated microwave power *P_micr_* on temperature: 1—of the epoxy basalt-filled oligomer; 2—of the epoxy oligomer ED-20.

Three characteristic areas corresponding to the EBO temperature after microwave processing were determined:

- The area of “non-thermal” effect corresponding to a slight heating of the EBO by 3–4 ° C at a low level of absorbed microwave power of 51 W (Figure 9, region I);
- The second characteristic area of the thermal effect corresponds to the level of the absorbed EBO power from 142 W to 239 W and a significant increase in temperature up to 190 °C (Figure 9, region II);
- The third area is the area of thermal destruction when the temperature approaches the decomposition temperature of the oligomer at the absorbed power level of more than 348 W and an increase in temperature above 230 °C. So, the mode of area III is technologically unacceptable (Figure 9, area III).

When a basalt filler is added into the plasticized epoxy oligomer, a thermal effect of a microwave EMF is observed in the range of 100–600 W, which is apparently associated with the chemical composition of basalt, namely, with a high content of metal oxides (Fe_2_O_3_, FeO, Al_2_O_3_, etc.), which results in the EBO heating with the effect of a microwave EMF.

### 3.2. The Physicochemical and Mechanical Properties of Epoxy Basalt-Filled Polymer Composite Material

As a result of the effect of microwave power on the physical and mechanical properties of EB PCM, an increase in impact strength by 2 times, breaking stresses in bending by 20%, tensile strength by 18% and hardness by 31% were noted (Table 4).

Thus, it was established that the highest values of impact strength, tensile strength and breaking stress in bending of the EB PCM are obtained when processed in a microwave EMF at *P_micr_* = 400 W, while the hardness value continues to grow and reaches a maximum value of 327 MPa at a power of 600 W. It should be noted that an increase in hardness above 300 MPa is accompanied by an increase in brittleness of the EB PCM.

The results of the study of the influence of the microwave processing duration on the physical and mechanical properties of the EB PCM at a power of 400 W show that the highest physical and mechanical characteristics are obtained with a microwave effect of 24 s (Table 5).

For PCM processed under the selected mode in a microwave EMF, the parameters of LOI and weight loss upon ignition in air practically do not change (Table 6), with a slight increase in heat resistance of 4.5% compared to the unmodified oligomer in a microwave EMF.

**Figure 10:**
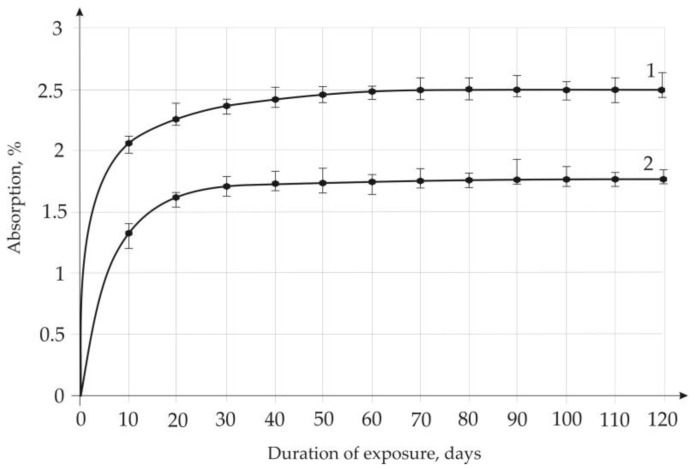
Changes in stability of the epoxy basalt-filled polymer composite material with 25% hydrochloric acid: 1—without microwave processing of the oligomer; 2—after microwave processing of the oligomer at *P_micr_* = 400 W, *τ_micr_* = 24 s.

**Figure 11:**
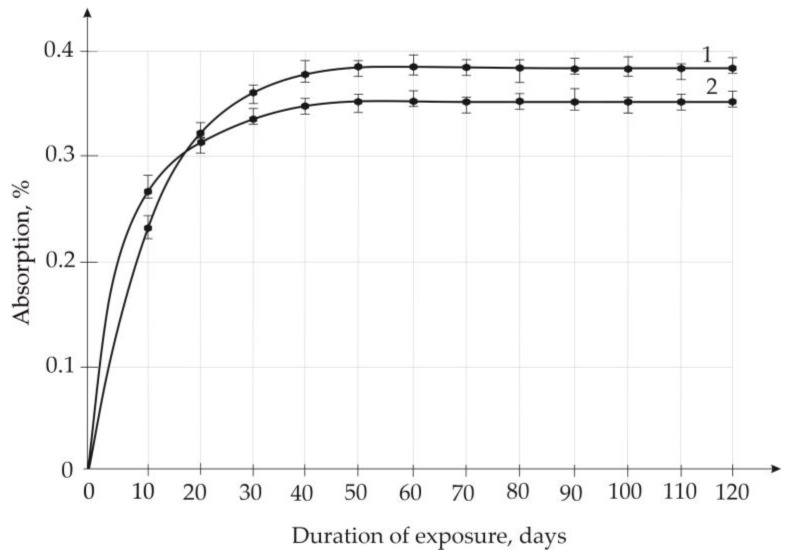
Changes in resistance of the epoxy basalt-filled polymer composite material with a 40% aqueous solution of sodium hydroxide: 1—without microwave processing; 2—after microwave processing of the oligomer at *P_micr_* = 400 W, *τ_micr_* = 24 s.

After microwave modification of the EBO under optimal processing conditions, an EB PCM having low water absorption values and high chemical resistance was obtained, (Table 7, Figure 10 and Figure 11), which indicated the formation of a denser, less defective structure. The indicators of resistance to various aggressive environments were studied.

Studies of changes in the properties of the EB PCM after testing showed that all physical and mechanical characteristics after exposure to an aggressive environment are reduced by less than 14% (Table 8), which corresponds to the good resistance of EB PCM to aggressive environments according to ISO 175:2010.

**Figure 12:**
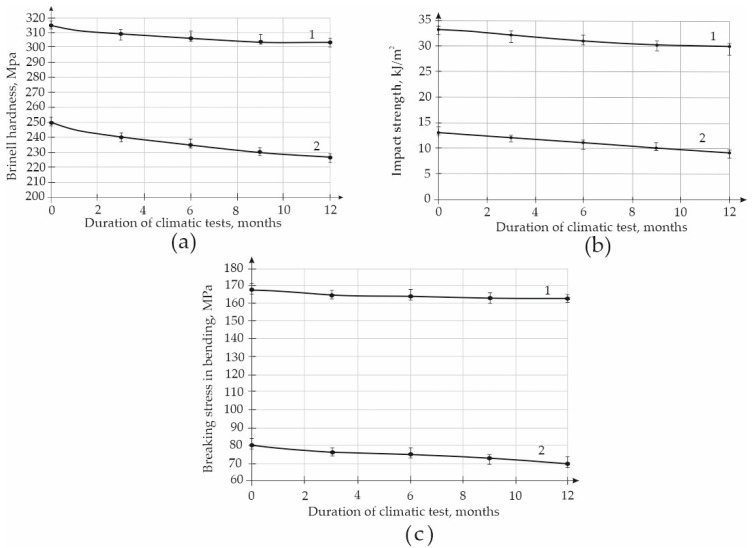
Influence of the duration of climatic impact on physical and mechanical properties ((**a**)—Brinell hardness; (**b**)—impact strength; (**c**)—breaking stresses in bending) of the epoxy basalt-filled polymer composite material: 1—after modification in a microwave EMF; 2—unmodified material.

For the EB PCM modified in a microwave EMF, an analysis of climatic resistance was carried out, which consisted of the impact of natural climatic factors during a given test duration (1, 3, 6, 9 and 12 months) in order to determine the resistance to the specified impact. The results show the change in the mechanical properties of the EB PCM under the impact of climate factors under natural conditions. A decrease in strength characteristics was established during exposure for 12 months of both the modified EB PCM in a microwave EMF and the unmodified one. At the same time, the coefficient of property retention for the unmodified EB PCM for impact strength was ~69%. For breaking stress in bending, it was 87.5%. For Brinell hardness, it was almost 90%, while for the microwave-processed composite material, the coefficient of property retention was above 90% for all physical and mechanical characteristics (Figure 12, Table 9). These results were confirmed by tests for water permeability and chemical resistance, which indicated a denser, less defective structure of the polymer composite material modified in a microwave EMF.

The results of climatic tests show that the most sensitive characteristic is impact strength (Table 9). Within 1 year of full-scale exposure, the value of impact strength for unmodified EB PCM decreases by 31% and by almost 10% for the microwave modified one—, which is associated with the effect of humidity, temperature and ultraviolet radiation on the polymer matrix of the EB PCM, causing its slow destruction (aging).

Thus, it was established that the effect of the microwave electromagnetic field on the EBO is an effective modification method that improves the physical and mechanical characteristics with a high level of their temporal stability. It does not reduce, and in some cases increases, the resistance of the EB PCM to climatic impact with the coefficient of property retention of more than 90%.

### 3.3. Influence of the Microwave Electromagnetic Field on the Structure of the Epoxy Basalt-Filled Oligomer

Since a microwave EMF affects the processes of structure formation, and accordingly, the properties of the EB PCM, the effect of a microwave EMF on the curing kinetics of EBO was studied (Table 10).

A study of the curing kinetics showed that branched macromolecules were formed in the EBO during curing within 42 min, with the completion of the curing reaction after 61 min at 87 °C. The effect of a microwave EMF on EBO reduces the gelation and curing time compared to the EBO without microwave exposure, which is associated with a decrease in the viscosity of the oligomer. The effect of microwave power on the degree of curing of the EBO with a PEPA hardener was established. The results show a complete EBO cross-linking, which is more than 97% at microwave power from 100 to 400 W. At high levels of microwave power, a decrease in the degree of curing was observed as a result of the start of the destruction process of the epoxy matrix.

**Figure 13:**
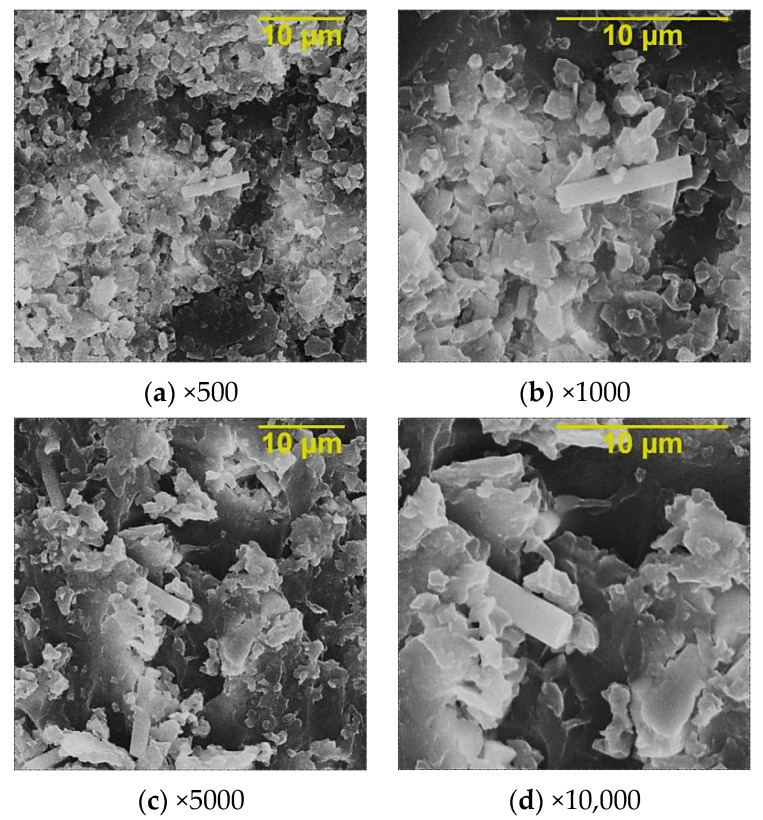
The results of scanning electron microscopy of images of a thin section of the EB PCM: (**a**,**b**) after microwave exposure; (**c**,**d**) without microwave exposure.

**Figure 14:**
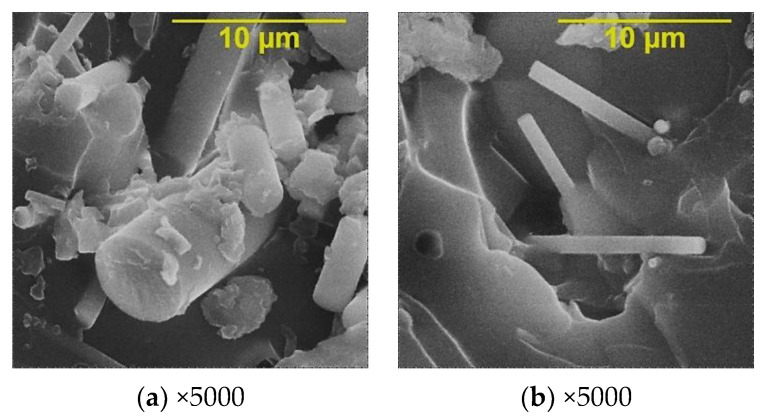
Results of scanning electron microscopy of images of the brittle chip on the EB PCM: (**a**) without microwave processing; (**b**) after microwave processing.

The results of scanning electron microscopy (Figure 13 and Figure 14) confirm the effect of a microwave EMF on the structure of the EB PCM obtained on the basis of a modified epoxy basalt-filled oligomer. When examining a thin section of the EB PCM (Figure 13), it was found that microwave exposure contributes to a more uniform distribution of fibrous and irregularly shaped basalt particles in the polymer matrix due to balancing the intermolecular bonds of the epoxy binder and filler (Figure 13a,b). At the same time (Figure 13c,d), large air inclusions of 10 µm or more were observed in the EB PCM obtained without microwave processing throughout the entire volume of PCM, as well as agglomerates of filler particles with a size of 20–30 µm.

The study of the brittle chip of the EB PCM is important for the development of the theory of the influence of a microwave EMF on the mechanism of the structural interaction between the epoxy matrix and the basalt filler (Figure 14) under the influence of a microwave EMF. The brittle fracture of the sample makes it possible to conditionally estimate the strength of the intermolecular interaction between the matrix and the filler. The original epoxy composition was characterized by brittleness, with a large number of elongated basalt fibers (Figure 14a), which do not allow achieving high strength indicators of the composite. Microwave processing of the epoxy oligomer reduces the viscosity of the composition, improving the impregnation of the surface of the filler, thereby improving the interfacial adhesion of the oligomer molecules with basalt particles. Moreover, it is reported in [35,36] that polar functional groups with active oxygen in the microwave field are locally heated more strongly than non-polar functional groups; therefore, microwave radiation can provoke interaction between polar groups at the phase boundary [37,38]. Thus, under the action of an external load on the epoxy matrix, it transfers this load to the filler more effectively due to stronger bonds at the phase boundary, thereby reducing the number of defects on the chipped surface. The improvement in interfacial interaction can be noted in a decrease in the number of basalt microfibers pulled out of the matrix and in the uniformity of the matrix at the chipped boundary (Figure 14b) [39,40].

**Figure 15:**
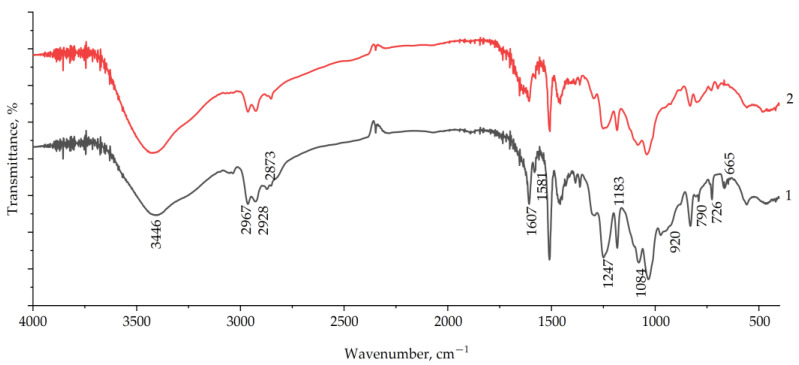
Results of IR spectroscopy of the EB PCM: 1—without microwave processing; 2—after microwave processing.

Analysis of the results of IR spectroscopy (Figure 15) showed the presence of OH groups (3446 cm^−1^), CH_2_ (2967 cm^−1^) and CH_3_ (2928–2873 cm^−1^) in the unmodified epoxy-basalt plastic, confirmed by the peaks of their deformation vibrations. Stretching vibrations of aromatic rings of epoxy resin (1607 cm^−1^, 1581 cm^−1^ and 920 cm^−1^), deformation (1247 cm^−1^ and 1183 cm^−1^) and stretching (1084 cm^-1^) vibrations of hydroxyl groups were determined. A weak maximum at 790 cm^−1^ was due to stretching vibrations of the Al-O bond; at 726 cm^−1^, it was due to vibrations of the -Si-O bond. A very weak maximum at 665 cm^−1^ reflected the stretching vibrations of the -Fe-O bond. These bonds are typical of the basalt filler. The IR spectrum of the modified EB PCM completely repeated the spectrum of the unmodified composite; however, the intensity of deformation vibrations of OH groups, epoxy groups and the -Si-O bond was reduced compared to the unmodified composite. This is likely due to a more complete intermolecular interaction of polar groups as a result of the microwave effect on epoxy resin, basalt and the plasticizer composing the EBO.

Analysis of the results obtained for heat resistance allows us to state that two intervals are observed under the influence of temperature:

- Volatilization of non-cross-linked epoxy groups;
- Decomposition of the high molecular weight fraction with the release of carbon monoxide, methane, ethane, ethylene, propylene, acetone, formaldehyde, acetaldehyde and benzene.

When studying thermal stability of epoxy-basalt plastics, a shift in the destruction stages to the region of higher temperatures is observed for composites obtained on the basis of a microwave modified oligomer, and an increase in their coke residues is also noted, which indicates their higher thermal stability compared to the unmodified composite (Table 11).

## 4. Conclusions

The results of the influence of the generated microwave power *P_micr_* on the power *R_abs_* absorbed by the EBO on the temperature of the oligomer after microwave exposure were obtained. The calculated values of the electric field strength E of the electromagnetic wave at which the maximum modifying effect is achieved were obtained as well, which is of great scientific and practical importance for the development of the oligomer microwave modification technology. The influence of the microwave electromagnetic field modes on the physical and mechanical properties of the epoxy basalt-filled polymer composite material was studied. We noted an increase in breaking stress in bending by 20%, impact strength by 2 times, hardness by 31% and a slight increase in heat resistance by 4.5% of the EB PCM compared to the composite obtained on the basis of an unmodified oligomer in a microwave EMF. At the same time, the highest values of the mechanical properties of the EB PCM were achieved when processed in a microwave EMF at a power of *P_micr_* = 400 W and an exposure duration of 24 s. The results of resistance to various aggressive environments were obtained, which show that the studied physical and mechanical characteristics of the EB PCM after exposure to an aggressive environment are reduced by less than 14%, which corresponds to good resistance of the EB PCM to aggressive environments.

The modifying effect of a microwave EMF on the oligomer was confirmed by the data of differential thermal analysis, scanning electron microscopy, the degree of curing of the basalt-filled epoxy oligomer, the results of IR spectroscopy and the change in the physical and mechanical properties of the EB PCM. The microwave modifying effect on the EBO is explained by the dipole-group polarization of the oligomer molecules along the force lines of the electric field strength of the electromagnetic wave, resulting in an increase in dielectric losses because the EMF energy is spent on the orientation of the dipoles, as well as on overcoming the forces of intermolecular interaction and alignment of the dipoles along the lines of force E and transformation into heat, heating the oligomer to 200 °C at a microwave power of 400 W.

As a result, there is a greater possibility to overcome the potential barrier of the rotation of the polar groups of the oligomer, which leads to an increase in kinetic flexibility of the polymer, which in turn creates additional possibilities for the formation of new intermolecular interactions of the epoxy and hydroxyl groups of the oligomer with the hydroxyl groups of basalt, as well as the C–Cl bond in TCEP with the OH group of basalt. High thermal stability of the modified EBO was established due to the shift of the destruction stages to the region of higher temperatures with an increase in their coke residues.

It was established that the impact of the microwave electromagnetic field on the EBO is an effective method of modification that improves the physical and mechanical characteristics, with a high level of temporal stability to climatic impact with a coefficient of property retention of more than 90%. The results of climatic tests show that the most sensitive characteristic is impact strength. During 1 year of full-scale exposure, the value of impact strength for the unmodified PCM EB was reduced by 31%; for the microwave modified one, it was reduced by 10%.

Thus, the effectiveness and expediency of using a microwave electromagnetic field for modifying a basalt-filled epoxy oligomer in order to improve physical and mechanical properties of the EB PCM based on it has been proven.

## Figures and Tables

**Table 1:** Chemical composition of basalt.

Component	Concentration, %
Silicon oxide (SiO_2_)	47–52
Titanium oxide (TiO_2_)	1–2.5
Aluminium oxide (Al_2_O_3_)	14–18
Iron oxide III (Fe_2_O_3_)	2–5
Iron oxide II (FeO)	6–10
Manganese oxide II (MnO)	0.1–0.2
Magnesium oxide (MgO)	5–7
Calcium oxide (CaO)	6–12
Sodium oxide (Na_2_O)	1.5–3
Potassium oxide (K_2_O)	0.1–1.5
Phosphorus oxide (P_2_O_5_)	0.2–0.5

**Table 2:** Main properties and characteristics of epoxy resin, TCEP, basalt and a hardener.

Qualitative Characteristics	Value
Properties of epoxy resin ED-20	
Density at 20 °C, kg/m^3^	1.166
Dynamic viscosity at 25 °C	13–20
Epoxy groups, %	20–22.5
Volatile substances and water, %	0.2
Gelation time, h	8
TCEP properties	
Molecular mass	285
Phosphorus content, %	10.9
Chlorine content, %	37
Boiling point, °C	106–108
Density, kg/m^3^	1068–1072
PEPA properties	
Mass fraction of nitrogen titrated with acid, %, no more	19
Water content, % no more	2
Density, g/cm^3^	0.956–1.011
Hardening capacity, h, no more	1.5
Basalt properties	
Density, kg/cm^3^	2520–2970
Melting point, °C	1100–1250
Water absorption, %	0.15–10.2
Specific heat capacity, at 0 °C, J/kg × K	0.84
Compression strength, MPa	400

**Table 3:** Parameters of microwave processing of the epoxy basalt-filled oligomer.

*P_micr_*, W	*P_abs_*, W/cm^3^	E, V/cm	T, °C	Effect
-	-	-	25	Non-thermal effect
100	51	50	40	
200	142	85	130	Thermal modifying effect
300	187	98	160	
400	239	119	190	
500	348	130	230	Thermal destructuring effect
600	351	131	250	

**Table 4:** Effect of microwave EMF power on the physical and mechanical properties of the epoxy basalt-filled polymer composite material.

Microwave Processing Power, W	Impact Strength *, kJ/m^2^	Breaking Stress in Bending, MPa	Brinell Hardness, MPa	Tensile Strength, MPa
-	13	122	250	32
100	18	134	262	32
200	22	137	275	34
300	24	140	284	36
400	31	152	298	38
500	26	144	308	36
600	20	135	327	35

Note: the coefficient of variation for properties is ~4–5%; *—samples were tested without a notch.

**Table 5:** The influence of the duration of microwave EMF exposure on the physical and mechanical properties of the epoxy basalt-filled polymer composite material.

Duration of Microwave Exposure, s	Impact Strength, kJ/m^2^	Breaking Stress in Bending, MPa	Brinell Hardness, MPa	Tensile Strength, MPa
31	31	152	298	38
27	33	158	306	39
24	37	167	314	42
21	35	162	310	40
19	30	153	309	38

Note: the coefficient of variation for properties is ~4–5%.

**Table 6:** Properties of the epoxy basalt-filled polymer composite material.

Processing Mode	Heat Resistance by Vicat, °C	LOI, % of Volume	Mass Loss at Ignition in Air, %
-	172	37	0.7
*P_micr_* = 400 W *Τ*_micr_ = 24 s	180	38	0.6

Note: the coefficient of variation for properties is ~4–5%.

**Table 7:** Indicators of resistance to aggressive environments of the epoxy basalt-filled polymer composite material.

Exposure Mode	Density, kg/m^3^	Water Absorption, %
-	1607	0.07
*P_micr_* = 400 W *τ_Cmicr_*= 24 s	1682	0.05

**Table 8:** The results of the influence of various environments on physical and mechanical characteristics of the epoxy basalt-filled polymer composite material after microwave processing of the EBO at *P_micr_* = 400 W, *τ_micr_* = 24 s.

Impact Environment	Impact Strength, kJ/m^2^	Breaking Stress in Bending, MPa	Brinell Hardness, MPa	Resistance Estimation According to ISO 175:2010
-	37	167	314	-
H_2_O	34	158	312	Good
HCl	30	146	278	Good
NaOH	30	142	290	Good

**Table 9:** The coefficient of properties retention (*K_t_*) of the epoxy basalt-filled polymer composite material under the impact of natural climatic factors.

Duration of Aging Tests on EB PCM under the Impact of Natural Climatic Factors, Months	*K_t_* for Impact Strength, %	*K_t_* for Breaking Stress in Bending, %	*K_t_* for Brinell Hardness, %
EBO without microwave processing + PEPA			
1	100	97.5	98.0
3	92.3	95.0	96.0
6	84.6	93.7	94.0
9	76.9	91.2	92.0
12	69.2	87.5	90.8
EBO processed in the microwave electromagnetic field + PEPA			
1	100	99.4	99.0
3	96.9	98.2	98.4
6	93.9	97.6	97.4
9	90.9	97.0	96.5
12	90.9	97.0	96.5

**Table 10:** Curing kinetics of the epoxy basalt-filled oligomer.

Kinetic Parameters	Without Microwave Exposure	After Microwave Exposure
Gelation time, min	70	42
Curing time, min	82	61
Maximum curing temperature, °C	85	87

**Table 11:** Effect of microwave processing on the heat resistance of epoxy basalt-filled polymer composite material.

Microwave Processing Parameters, W	Parameters of Destruction Stages		Mass Loss %, at Different Temperatures, °C						Coke Residues at 700 °C, %
	T_h_-T_k_, °C T_max_	m_h_-m_k_, % m_max_	200	300	400	500	600	700	
-	210–288 254 338–644 511	8–15 13 15–38 29	7	16	21	29	38	38	62
*P_micr_* = 400 W *τ_micr_* = 24 s	217–292 266 352–650 526	7–12 10 13–30 22	7	12	17	24	29	33	67
